# *CFH* (rs1061170, rs1410996), *KDR* (rs2071559, rs1870377) and KDR and CFH Serum Levels in AMD Development and Treatment Efficacy

**DOI:** 10.3390/biomedicines12050948

**Published:** 2024-04-24

**Authors:** Dzastina Cebatoriene, Alvita Vilkeviciute, Greta Gedvilaite, Akvile Bruzaite, Loresa Kriauciuniene, Dalia Zaliuniene, Rasa Liutkeviciene

**Affiliations:** 1 Medical Academy, Lithuanian University of Health Sciences, A. Mickeviciaus St. 9, LT-44307 Kaunas, Lithuania; 2 Neuroscience Institute, Medical Academy, Lithuanian University of Health Sciences, Eiveniu St. 2, LT-50161 Kaunas, Lithuania; alvita.vilkeviciute@lsmu.lt (A.V.); greta.gedvilaite@lsmu.lt (G.G.); akvile.bruzaite@lsmu.lt (A.B.); loresa.kriauciuniene@lsmu.lt (L.K.); rasa.liutkeviciene@lsmu.lt (R.L.); 3 Department of Ophthalmology, Medical Academy, Lithuanian University of Health Sciences, Eiveniu St. 2, LT-50161 Kaunas, Lithuania; dalia.zaliuniene@lsmu.lt

**Keywords:** age-related macular degeneration, gene polymorphisms KDR, CFH, ELISA, anti-VEGF therapy

## Abstract

Background: Age-related macular degeneration (AMD) is a major global health problem as it is the leading cause of irreversible loss of central vision in the aging population. Av-vascular endothelial growth factor (anti-VEGF) therapies have been shown to be effective, but they do not respond optimally to all patients. Objective. This study investigates the genetic factors associated with susceptibility to AMD and response to treatment, focusing on key polymorphisms in the *CFH* (rs1061170, rs1410996) and *KDR* (rs2071559, rs1870377) genes and the association of CFH and KDR serum levels in patients with AMD. Results. A cohort of 255 patients with early AMD, 252 patients with exudative AMD, and 349 healthy controls underwent genotyping analysis, which revealed significant associations between CFH polymorphisms and the risk of exudative AMD. The *CFH* rs1061170 CC genotype was associated with an increased risk of early AMD (*p* = 0.046). For exudative AMD, the *CFH* rs1061170 TC + CC genotype increased odds (*p* < 0.001), while the rs1410996 GA + AA genotype decreased odds (*p* < 0.001). Haplotypes of *CFH* SNPs were associated with decreased odds of AMD. In terms of response to treatment, none of the SNPs were associated with the response to anti-VEGF treatment. We also found that both early and exudative AMD patients had lower CFH serum levels compared to the control group (*p* = 0.038 and *p* = 0.006, respectively). Exudative AMD patients with the CT genotype of *CFH* rs1061170 had lower CFH serum levels compared to the control group (*p* = 0.035). Exudative AMD patients with the GG genotype of *CFH* rs1410996 also had lower CFH serum levels compared to the control group (*p* = 0.021). Conclusions. *CFH* polymorphisms influence susceptibility to AMD but do not correlate with a response to anti-VEGF therapy. Further research is imperative to fully evaluate the developmental significance, treatment efficacy, and predictive role in influencing susceptibility to anti-VEGF therapy for KDR and CFH.

## 1. Introduction

Age-related macular degeneration (AMD) is the most common cause of irreversible loss of central vision in the older population of the industrialized world [1,2,3]. Accordingly, it increases the demands on the healthcare system as people live longer and treatment costs rise exponentially. AMD is responsible for around 9% of all blindness [4]. This disease affects the back of the eye and damages the macula; a part of the retina with many photoreceptor cells that are responsible for central vision [5]. Aging contributes significantly to AMD, and the age-adjusted prevalence is around 24% in people aged 65–74 years and more than 44% in 70–95-year-olds [6,7]. The human retina undergoes changes as a natural part of aging. This process results in the formation of visible focal yellow deposits called drusen, which consist of cellular debris between the retinal pigment epithelium and Bruch’s membrane. When these pathophysiological changes impair vision, and are accompanied by hypo- or hyper-pigmentation of retinal pigment epithelial (RPE) cells and enlargement or increasing confluence of drusen, this is diagnosed as AMD [8].

However, there is no treatment for the progression of dry AMD or GA so far [9]. The “wet” or neovascular form is less common but is responsible for 90% of acute blindness due to AMD [10], and the prevalence of advanced AMD is estimated at 1.6% worldwide [1]. Neovascular AMD (nAMD) is characterized by the formation of neovascular choroidal membranes, exudation, and fibrosis leading to acute vision loss [11].

Most therapies for the treatment of nAMD target the abnormal growth of blood vessels by inhibiting vascular endothelial growth factor (VEGF)-based antibodies, showing a range of effects. Significant strides have been made in the treatment of nAMD since the era of thermal laser photocoagulation and photodynamic therapy two to three decades ago [12]. The gold standard therapy for maintaining or improving visual acuity in most patients with nAMD is now intravitreal injection of anti-vascular endothelial growth factor. Many studies have shown positive results with anti-VEGF drugs, but there are also limitations to their use. Previous studies have shown that 20% of patients still lose their vision, and half of patients ultimately fail to achieve 20/40 visual acuity [13,14]. A large published meta-analysis showed that visual acuity with best-corrected visual acuity in 80-year-olds with nAMD has improved significantly since 2006 [15]. The improvement in visual acuity is thought to be related to a healthier lifestyle and the introduction of anti-vascular endothelial growth factor therapy. By default, the response to anti-VEGF therapy can be monitored by evaluating visual acuity and optical coherence tomography parameters such as central retinal thickness (CRT) or total macular volume, which reflect the extent of edema. Nevertheless, despite these therapies, most patients require indefinite treatment, do not regain their vision, or show progression of the disease [16]. However, there remains an unmet clinical need for new and improved therapies for nAMD, as many patients do not respond optimally to treatment, and the effect diminishes over time or has suboptimal durability, which compromises efficacy in practice. There is evidence that targeting VEGF-A alone, as has been the case with most agents until recently, may be insufficient and that agents targeting multiple signaling pathways (e.g., aflibercept, faricimab, and other agents in development) may be more effective.

Although genes may only have a minor impact on the total genetic variance of AMD, their effects do not consistently align with the significance of the disease’s pathogenesis and subsequent treatment [17]. It is important to note that the percentage of cases attributed to specific genetic variants does not necessarily indicate the genes’ role in the disease’s pathophysiology [17]. Therefore, the search for other existing loci that require clarification should be continued [18,19,20,21,22,23,24,25,26,27]. In the case of the GWAS experiment [28], genes were found that not only function in known AMD signaling pathways but also reveal the importance of additional pathways, including complement activation, collagen synthesis, lipid metabolism/cholesterol transport, receptor-mediated endocytosis, endodermal cell differentiation, and extracellular matrix organization. The VEGFR-2 receptor, encoded by the *KDR* gene, serves as a high-affinity receptor tyrosine kinase that is responsible for the majority of angiogenic and permeability-enhancing effects induced by VEGF-A. Consequently, *KDR* variants are considered potential candidates for influencing sensitivity to anti-VEGF therapy [29]. Research conducted by Lazzeri et al. [29,30,31] indicates that the *KDR* (*VEGFR-2*) genotype rs2071559 might serve as a predictive factor for both short- and long-term functional and anatomical outcomes in individuals with nAMD undergoing treatment with ranibizumab. In addition, Hermann et al. [30] concluded that genetic polymorphisms in the *KDR* gene play an important role in influencing the visual outcomes of patients treated with ranibizumab for nAMD. Ranibizumab binds VEGF-A and inhibits its effect on VEGFR-1 and -2. VEGFR-1 and -2 are encoded by FLT1 and KDR, respectively. In the context of ranibizumab binding to VEGFA, these variants may cause conformational changes or alter VEGFR1 delivery and expression. Consequently, these genetic variations may contribute to individual differences in response to ranibizumab. Recently, great progress has been made in the pathology and epidemiology of AMD, particularly in the field of genetics. Several genes have been identified that are associated with susceptibility to AMD, with the complement factor H (*CFH*) gene being one of the most important [32]. A meta-analysis of the Asian population found that the *CFH* polymorphisms rs1061170 and rs1410996 were associated with AMD risk, with both demonstrating higher susceptibility to AMD, particularly nAMD [33]. Whole genome sequencing identifies a significant association between *CFH* loci with AMD [34].

All in all, AMD is a multifactorial disease and identifying risk factors allows individuals to make lifestyle choices that can reduce their risk of developing the disease. In this study, we intentionally focused on investigating the association between specific polymorphisms and the response to AMD therapy. While diagnostic accuracy could provide valuable insights, our study was designed to prioritize understanding the genetic factors impacting therapeutic outcomes in AMD management. Therefore, our primary objective was to elucidate how genetic variations influence the effectiveness of AMD treatment modalities.

## 2. Materials and Methods

### 2.1. Study Design and Structure

The current study was conducted according to the guidelines of the Declaration of Helsinki, and the protocol was approved by the Kaunas Regional Biomedical Research Ethics Committee, Lithuanian University of Health Sciences (No. BE-2-/48). All study participants signed the informed consent form. An ophthalmological evaluation was performed for all the study subjects admitted for ophthalmological assessment at the ophthalmology department, Hospital of Lithuanian University of Health Sciences, from 2018 to 2023. Their health and other diseases were obtained during the general practitioner examination and gathered from medical records.

Our study involved 255 patients diagnosed with early AMD, 252 patients with exudative AMD, and 349 healthy controls. The control group was formed of 349 subjects that matched gender classification in the early and exudative AMD group structure; however, subjects of the control group were younger than exudative AMD patients (*p* < 0.001), and further analysis was performed adjusted by age (Table 1).

AMD classification is defined by the American Academy of Ophthalmology as follows:

- Early AMD: Defined by the presence of numerous small (<63 microns, “hard”) or intermediate (≥63 microns but <125 microns, “soft”) drusen.
- Intermediate AMD: Macular disease characterized by either extensive drusen of small or intermediate size, or any drusen of large size (≥125 microns).
- Advanced AMD: Defined by the presence of either geographic atrophy or choroidal neovascular membrane (along with its sequelae, such as subretinal or sub-RPE hemorrhage or serous fluid, and subretinal fibrosis).

The AMD group consisted of subjects aged 55 years or older who underwent ophthalmological evaluation and were diagnosed with early, exudative AMD.

All patients underwent a comprehensive eye examination including best corrected visual acuity (BCVA) using the ETDRS visual chart, retinal photography of the fundus, structural OCT (Triton SS-OCT, Topcon, Tokyo, Japan), and OCT angiography (OCT-A). Fluorescein angiograms were performed if necessary.

Patients received a loading dose of 3-monthly injections of anti-VEGF and were then followed up monthly; treatment intervals (2 to 4 weeks) were shortened when disease activity recurred. The exudative AMD group consisted of patients with active subfoveal CNV lesions (SRS, IRS fluid, or macular thickening) caused by AMD. Juxtafoveal lesions with leakage affecting the fovea were also included.

The early AMD group consisted of patients with numerous small “hard” or intermediate “soft” drusen in the early stages of AMD.

AMD exclusion criteria consisted of the following:

- Unrelated eye disorders; e.g., high refractive error, cloudy cornea, lens opacity (nuclear, cortical, or posterior subcapsular cataract) except minor opacities, keratitis, acute or chronic uveitis;
- Systemic illnesses; e.g., diabetes mellitus, malignant tumors, systemic connective tissue disorders, chronic infectious and non-infectious diseases, coronary artery disease, stroke, or conditions following organ or tissue transplantation;
- Ungraded color fundus photographs resulting from obscuring the ocular optic system or because of fundus photograph quality;
- Use of antiepileptic or sedative drugs.

Based on the clinical OCT and BCVA data, patients with exudative AMD were categorized into one of two groups: responders and non-responders.

The efficacy of anti-VEGF treatment (ranibizumab, aflibercept, bevacizumab) was evaluated in patients with exudative AMD who had exudative or hemorrhagic features in the macula, but had not received a prior intravitreal anti-VEGF injection or other treatment and were followed up for at least 6 months after the first anti-VEGF injection. Central macular thickness (CMT) and best corrected visual acuity (BCVA) were measured before treatment and six months after the first intravitreal anti-VEGF injection.

Visual acuity was assessed before treatment and six months after the first intravitreal anti-VEGF injection. Deterioration in visual acuity was considered to have occurred if patients had lost one or more line (>5 letters) in the table. BCVA changes during the treatment period were calculated using the following formula: BCVA after six months minus BCVA before treatment.

Good response was defined when there was resolution of fluid according to OCT 6 months after the first injection, and/or improvement of >5 letters.

Non-response was defined as an increase in fluid of 100 μM (IRF, SRF and CRT), or increasing hemorrhage compared with the baseline and/or loss of >5 letters compared with the baseline or best corrected vision subsequently. CMT changes were calculated accordingly: CMT before treatment minus CMT after 6 months.

Subjects who underwent ophthalmological evaluation were involved in the control group.

Control group inclusion criteria consisted of the following:

- Older than 18 years;
- Patients after senile cataract surgeries (without any other ocular comorbidities);
- Signed informed consent form.

Control group exclusion criteria consisted of the following:

- Unrelated eye disorders; e.g., high refractive error, cloudy cornea, lens opacity (nuclear, cortical, or posterior subcapsular cataract) except minor opacities, keratitis, acute or chronic uveitis, glaucoma, or diseases of the optic nerve;
- Systemic illnesses; e.g., diabetes mellitus, malignant tumors, systemic connective tissue disorders, chronic infectious and non-infectious diseases, hypertension, coronary artery disease, stroke, or conditions following organ or tissue transplantation;
- Ungraded color fundus photographs resulting from obscuring the ocular optic system or because of fundus photograph quality;
- Use of antiepileptic or sedative drugs.

### 2.2. SNP Selection

Our selection aimed to encompass variants with established relevance to AMD pathogenesis and treatment response.

In our study, the selection of SNPs was based on previous literature indicating their associations with exudative AMD occurrence and/or response to anti-VEGF injections. Specifically, we selected four SNPs from the *CFH* and *KDR* genes. From the CFH gene, we selected rs1061170 and rs1410996 based on their documented associations with response to anti-VEGF treatment in AMD patients [35].

KDR rs2071559 was chosen due to its association with AMD occurrence in previous research [29,30,31]. The KDR rs1870377 was selected as a potential biomarker for AMD treatment response even if the link between it and exudative AMD was not confirmed yet, but the associations with other diseases and their treatment were significant in several studies [36,37].

### 2.3. Deoxyribonucleic Acid Extraction from Peripheral Venous Blood and Genotyping

Deoxyribonucleic acid (DNA) extraction and genotyping of selected single nucleotide polymorphisms (SNPs), *CFH* (rs1061170, rs1410996) and *KDR* (rs2071559, rs1870377), were conducted at the Laboratory of Ophthalmology, Neuroscience Institute, Lithuanian University of Health Sciences. Utilizing predesigned TaqManTM genotyping assays from Thermo Fisher Scientific, Pleasanton, CA, USA, the procedures were executed in accordance with the manufacturer’s instructions, following established protocols. To ensure high-quality DNA samples, the DNA salting-out method was selected for DNA extraction.

SNPs were determined using TaqMan^®^ genotyping assays (Applied Biosystems, New York, NY, USA; Thermo Fisher Scientific, Inc., Waltham, MA, USA), C___8355565_10, C___2530294_10, C__15869271_10 and C__11895315_20 according to manufacturer’s protocols by a StepOne Plus (Applied Biosystems, Waltham, MA, USA). For each reaction, 1 μL of genomic test DNA and 9 μL of PCR reaction mix were used. The composition of the PCR mixture and the PCR reaction conditions are given in Table 2 and Table 3, respectively.

### 2.4. Serum Protein Concentration Measurement

To prepare the serum, peripheral venous blood was collected and incubated for 30 min at room temperature before centrifugation. After centrifugation, the serum was separated from the pellet, transferred into 2 mL tubes, and then frozen at −80 °C until analysis. Serum KDR levels in AMD patients and control subjects were assessed following the manufacturer’s guidelines. The analysis utilized an KDR ELISA Kit (Human) (Aviva Systems Biology, San Diego, CA, USA), employing standard sandwich ELISA technology with a range of 0.78–50 ng/mL. Similarly, serum CFH levels were determined in AMD patients and control subjects using an Invitrogen Complement Factor H ELISA Kit (Human) (Thermo Fisher Scientific, United States), which operates on a sandwich-type principle with a range of 2–500 ng/mL, as per the manufacturer’s instructions.

### 2.5. Statistical Analysis

The statistical analysis was carried out using SPSS/W 29.0 software (Statistical Package for the Social Sciences for Windows, Inc., Chicago, IL, USA). Continuous data (age, BCVA and CMT) were tested for normality using the Shapiro–Wilk test. Continuous variables were presented as median with interquartile range (IQR) and compared using a non-parametric Mann–Whitney U test. Wilcoxon signed rank test was used to evaluate the BCVA and CRT changes after the treatment with anti-VEGF. Statistically significant differences were observed when *p* < 0.05.

Categorical data (sex and genotype distributions) are presented as absolute numbers with percentages in parentheses and compared between groups using the chi-squared test (χ2).

The effect of gene polymorphisms on early and exudative was assessed using binomial logistic regression analysis and presented as an odds ratio (OR) with a 95% confidence interval (CI) after adjusting for sex in early AMD and age in the exudative AMD groups. The results of the logistic regression analysis were presented as genetic models (codominant: heterozygotes versus wild-type homozygotes and minor allele homozygotes versus wild-type homozygotes; dominant: minor allele homozygotes and heterozygotes versus wild-type homozygotes; recessive: minor allele homozygotes versus wild-type homozygotes and heterozygotes; overdominant: heterozygotes versus wild-type homozygotes and minor allele homozygotes; the additive model was used to assess the effects of each minor allele on AMD). The selection of the best genetic model was based on the Akaike information criterion (AIC); therefore, the best genetic models had the lowest AIC values. Due to the multiple association calculations, we introduced a Bonferroni correction and applied an adjusted significance threshold for multiple comparisons α = 0.0125 (0.05/4, as we analyzed four different SNPs).

## 3. Results

### 3.1. Hardy–Weinberg Equilibrium Analysis

Our study quality assessment based on Hardy–Weinberg equilibrium (HWE) analysis showed that the distribution of genotypes of *KDR* (rs2071559, rs1870377), *CFH* (rs1061170, rs1410996) did not deviate from HWE in the control group (*p* < 0.05).

### 3.2. Analysis of KDR (rs2071559, rs1870377), CFH (rs1061170, rs1410996) in Early and Exudative AMD

Our study genotyping data showed that there were no statistically significant differences in genotype and allele distributions of *KDR* (rs2071559 and rs1870377) between early AMD and control groups, or between exudative AMD and controls, while the analysis of *CFH* rs1061170 and rs1410996 genotype and allele distributions revealed significant results. *CFH* rs1061170 genotypes (TT, TC, and CC) and rs1410996 genotypes (GG, GA and AA) were distributed significantly differently when comparing patients with exudative AMD and control subjects (15.1%, 51.6% and 33.3% vs. 39.3%, 45.3% and 15.5%, *p* < 0.001 and 64.3%, 32.5% and 3.2% vs. 37.8%, 48.1% and 14%, *p* < 0.001, respectively) (Table 4). Further analysis showed that the C allele at *CFH* rs1061170 was more frequent in the exudative AMD group than in the control group (59.1% vs. 38.1%, respectively, *p* < 0.001) and the A allele at rs1410996 was significantly less frequent in exudative AMD group than in controls (19.4% vs. 38.1%, respectively, *p* < 0.001) (Table 4).

Binary logistic regression analysis was performed to evaluate the impact of SNPs on early and exudative AMD. It showed that *CFH* rs1061170 TC + CC genotypes were associated with increased odds of early AMD under the dominant (OR = 1.414; CI: 1.005; 1.988; *p* = 0.046) genetic model, but these results did not survive after Bonferroni correction (Table 5). Also, *CFH* rs1061170 variant showed significant associations with increased odds of exudative AMD occurrence under the codominant (OR = 2.961; CI: 1.894; 4.630, *p* < 0.001 and OR = 5.578; CI: 3.319; 9.377; *p* < 0.001), dominant (OR = 3.629; CI: 2.372; 5.552; *p* < 0.001), recessive (OR = 2.704; CI: 1.796; 4.071; *p* < 0.001) and additive (OR = 2.354; CI: 1.820; 3.046; *p* < 0.001) genetic models after adjustment for age. Moreover, we found that *CFH* rs1410996 was associated with highly decreased odds of exudative AMD occurrence under the codominant (OR = 0.380; CI: 0.262; 0.550; *p* < 0.001 and OR = 0.119; CI: 0.053; 0.266; *p* < 0.001), dominant (OR = 0.318; CI: 0.223; 0.453; *p* < 0.001), recessive (OR = 0.184; CI: 0.084; 0.403; *p* < 0.001), overdominant (OR = 0.509; CI: 0.357; 0.725; *p* < 0.001) and additive (OR = 0.363; CI: 0.270; 0.488; *p* < 0.001) genetic models after the same adjustment for age (Table 5).

### 3.3. Analysis of KDR (rs2071559, rs1870377) and CFH (rs1061170, rs1410996) in Early and Exudative AMD in Male and Female Subgroups

We found that *KDR* rs2071559 GA genotype is associated with decreased odds of early AMD in men under the codominant genetic model (OR = 0.491; 95% CI: 0.254–0.946; *p* = 0.033).

Also, GA and AA genotypes together are associated with similarly decreased odds of early AMD in men under the dominant genetic model (OR = 0.491; 95% CI: 0.254–0.946; *p* = 0.033). Further analysis showed that the KDR rs2071550 GA genotype is associated with 2-fold decreased odds of exudative AMD in men under the codominant genetic model (OR = 0.500; 95% CI: 0.266–0.940; *p* = 0.031). Also, GA and AA genotypes together are associated with similarly decreased odds of exudative AMD in men under the dominant genetic model (OR = 0.508; 95% CI: 0.279–0.925; *p* = 0.027). However, the results remained insignificant after Bonferroni correction (Table 6).

Statistical analysis for *CFH* rs1061170 and rs1410996 in subgroups by gender showed the same results as in the overall group: *CFH* rs1061170 is associated with increased odds of exudative AMD and the *CFH* rs1410996 was associated with the decreased odds of exudative AMD in males and females, and these results remained significant even after strict Bonferroni correction (Table 6).

### 3.4. KDR and CFH Haplotype Associations with AMD

A strong pairwise linkage disequilibrium (LD) was observed between the polymorphisms *CFH* rs1061170 and rs1410996 (Table 7).

We identified *KDR* and *CFH* haplotypes and analyzed their frequencies between the early and exudative AMD and control. The results of frequencies of haplotypes have shown that haplotypes of *CFH* SNPs (rs1061170C-rs1410996G and rs1061170T-rs1410996A) are associated with the decreased odds of early (OR  =  0.76; 95% CI: 0.58–1.00; *p* = 0.049 and OR  =  0.60; 95% CI: 0.45–0.82; *p* = 0.0011, respectively) and exudative (OR  =  0.29; 95% CI: 0.20–0.40; *p* < 0.001 and OR  =  0.42; 95% CI: 0.30–0.58; *p* < 0.001, respectively) AMD. The *CFH* haplotype rs1061170C-rs1410996A showed an association with decreased odds of exudative AMD (OR = 0.03; 95% CI: 0.00–0.21; *p* < 0.001). Analysis of KDR haplotypes did not show any associations with early or exudative AMD (Table 8).

### 3.5. Serum KDR and CFH Associations with AMD

**Figure 1:**
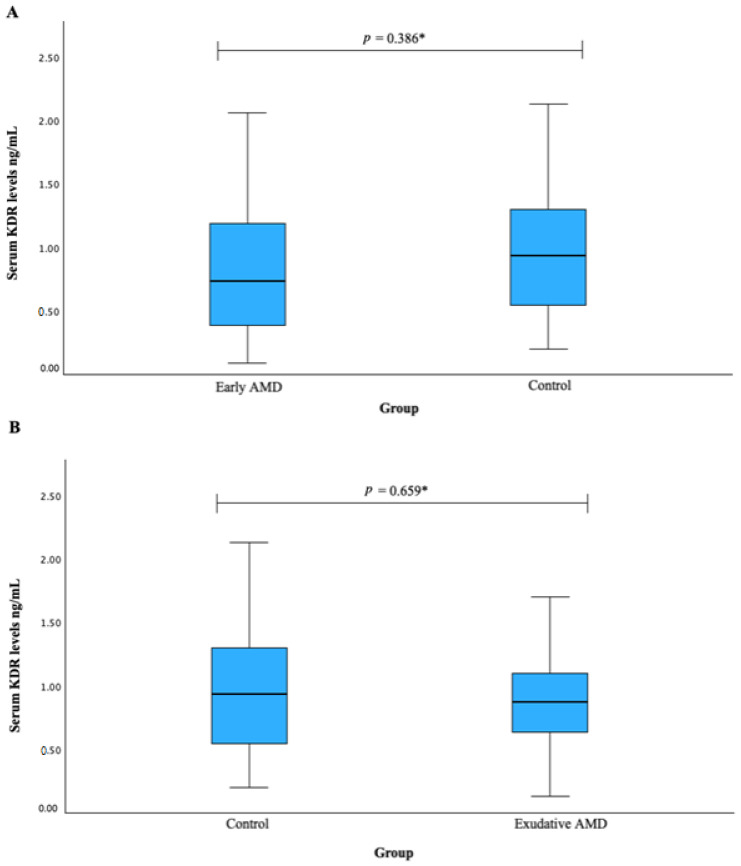
Serum KDR levels were measured in patients with early AMD vs. control group (**A**) and exudative AMD vs. control groups (**B**). * Mann–Whitney U test was used.

Serum KDR levels were measured in patients with early AMD vs. control group (A) and exudative AMD vs. control groups; however, no statistically significant difference was found (median (IQR): 0.732 (0.840) vs. 0.938 (0.771), *p* = 0.386; median (IQR): 0.871 (0.500) vs. 0.938 (0.771), *p* = 0.659, respectively). The results are shown in Figure 1.

**Figure 2:**
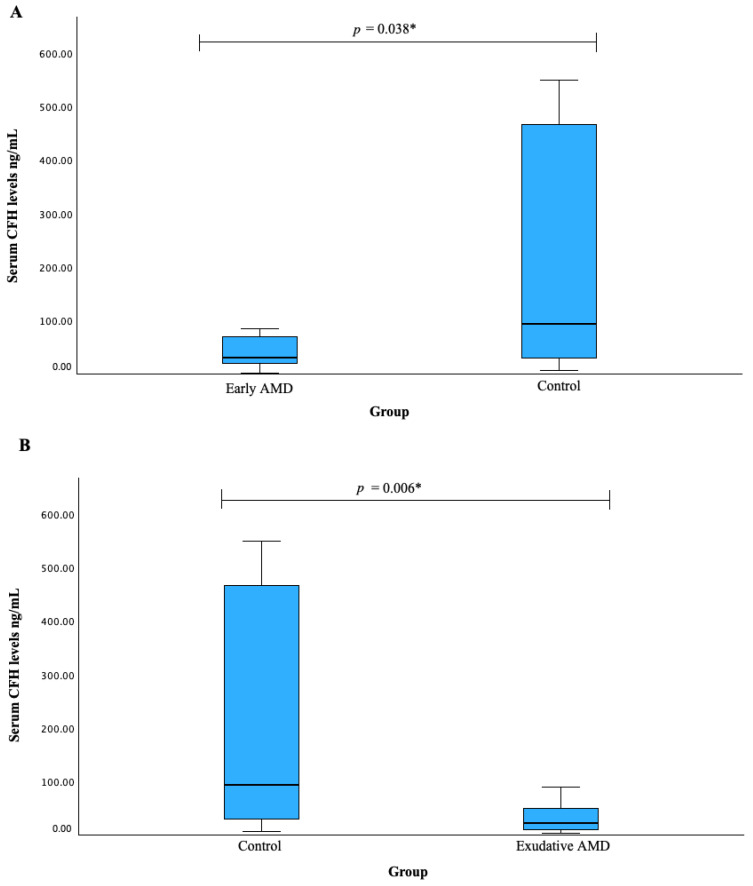
Serum CFH levels were measured in patients with early AMD vs. control group (**A**) and exudative AMD vs. control groups (**B**). * Mann–Whitney U test was used.

Serum CFH levels were measured in patients with early AMD vs. control group (A) and exudative AMD vs. control group (B). We found that both early and exudative AMD patients had decreased CFH serum levels when compared to the control group subjects (median (IQR): 29.866 (53.707) vs. 93.550 (443.224), *p* = 0.038; median (IQR): 21.437 (42.549) vs. 93.550 (443.224), *p* = 0.006, respectively). The results are shown in Figure 2.

### 3.6. Serum CFH Levels and CFH SNPs Associations with AMD

**Figure 3:**
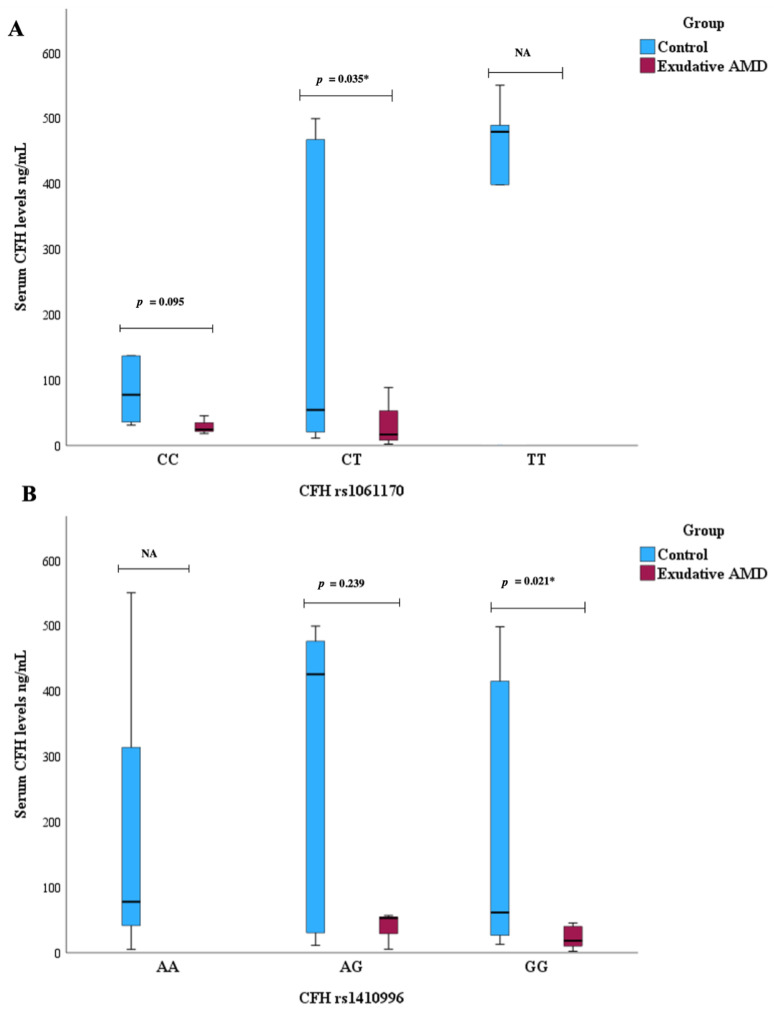
Serum CFH levels were measured in patients with exudative AMD vs. control group and compared between *CFH* rs1061170 genotypes (**A**) and between *CFH* rs1420996 genotypes (**B**). * Mann–Whitney U test was used.

A comparison of serum CFH levels was conducted among different genotypes for selected single nucleotide polymorphisms. Exudative AMD patients with the CT genotype of *CFH* rs1061170 exhibited lower serum CFH levels compared to the control group (median (IQR): 16.89 (48.22) vs. 54.32 (448.94), *p* = 0.035 (Figure 3A).

Exudative AMD patients with the GG genotype of *CFH* rs1410996 showed lower serum CFH levels compared to the control group, median (IQR): 18.48 (33.76) vs. 61.44 (417.34), *p* = 0.021) (Figure 3B).

No *CFH* level and *CFH* rs1061170 and rs1410996 genotype associations were revealed with early AMD occurrence.

### 3.7. Response to Exudative AMD Treatment with Anti-VEGF Therapy

The response to treatment was assessed in 121 individuals who had exudative age-related macular degeneration. The demographic and response parameters of the study population are summarized in Table 9. There was no difference in age or gender distribution between non-responders and responders.

The median visual acuity (VA) decreased in non-responders after treatment but increased in the responders group: (0.42 (0.45) vs. 0.31 (0.31), *p* = 0.020 and 0.26 (0.7) vs. 0.35 (0.32), *p* < 0.001, respectively). On the other hand, the CRT did not change statistically significantly in non-responders after treatment but was significantly thinner in responders after treatment (272.5 (86.5) vs. 314.5 (87.75) *p* = 0.386) and 321 (114) vs. 275 (94), *p* < 0.001, respectively) (Table 9).

### 3.8. Genetic Associations with Exudative AMD Response

Statistical analysis was performed to analyze the association between all four SNPs and treatment response. However, none of these SNPs were found to be linked to treatment response with anti-VEGF therapy.

### 3.9. Serum KDR and CFH Associations with Exudative AMD Response

A comparison of serum KDR and CFH levels was conducted among non-responders and responders, but no statistically significant differences were observed comparing these groups (*p* > 0.05).

## 4. Discussion

AMD affects around 170 million people worldwide, making it the third most common cause of visual impairment [35,38,39]. Clinically, AMD is divided into early stage (medium-sized drusen and pigmentary changes in the retina) and late stage (neovascular and atrophic) [5]. Early AMD is usually asymptomatic and can lead to a mild loss of visual acuity and function that delays the onset of night blindness [8]. In intermediate dry AMD, some people have no symptoms at first, but some notice mild symptoms such as a slight blurring of central vision or difficulty seeing in dim light. In late AMD (wet or dry type), many people complain of wavy or crooked lines, notice a blurred area near the center of vision, or see blank spots. Colors may also appear less vivid, and patients may have more difficulty seeing them in low-light conditions. Geographic atrophy is described as bilateral but not symmetrical. It is an area where there is a loss of the RPE and choriocapillaris with a corresponding loss or dysfunction of the overlying photoreceptors, and vision is dramatically impaired. GA affects more than 5 million people worldwide, and its prevalence in one eye is reported to be 0.6% [1].

It is known that Anti-VEGF treatment is one of the first therapies to benefit many AMD patients. Despite the efficacy of anti-VEGF drugs in many patients, some patients do not fully respond to treatment, and persistent intraretinal or subretinal fluid and vision loss occur. Patients with AMD have a reduced quality of life as several daily activities require functional central visual perception, such as driving and reading [40]. The findings from the 2-year analysis of AMD treatment studies revealed that 51.5% of eyes receiving monthly ranibizumab treatment and 67.4% of those receiving monthly bevacizumab treatment still exhibited persistent fluid on optical coherence tomography. Retrospective investigations into intravitreal ranibizumab therapy for neovascular AMD patients demonstrated recurrence rates ranging from 66% to 76% after 12 months of repeated treatment, and 74.8% after 24 months of treatment [41]. Recent research suggests that patients undergoing repeated intravitreal aflibercept injections for the treatment of neovascular AMD may experience disease recurrence in the range of 9% to 55%. This emphasizes the emergence of acquired resistance to anti-VEGF therapy [42]. An essential next step is to understand the functional consequences and downstream effects of AMD-associated genetic variants. Many genes that are considered risk factors for AMD are single nucleotide polymorphisms (SNPs), where one amino acid within the protein is replaced by another. SNPs can have different consequences. Those mutations in which a similar amino acid within a protein is replaced, e.g., valine by alanine, lead to a minor change in the protein [43]. Over the past decade, collaboration between geneticists and ophthalmologists has provided strong evidence that genetic factors are involved in AMD [44,45,46,47]; the changes have been found primarily in genes involved in immune modulation and the complement system—which plays an important role in the disease—along with other risk factors such as smoking [48,49,50,51], diet [52,53,54,55,56,57] and sun exposure [58,59]. Studies have shown that exposure to blue light emitted by smartphones and other devices damages vision and increases the risk of blindness [60]. Exposure of the retina to light from these devices promotes the formation of toxic molecules in the cells (photoreceptors or non-photoreceptors), which increases the risk of macular degeneration.

In our study, four SNPs in the *CFH* (rs1061170, rs1410996) and *KDR* (rs2071559, rs1870377) genes, and response to treatment of exudative AMD were investigated, but none of the SNPs were found to be associated with response to anti-VEGF therapy. Numerous clinical and observational studies have repeatedly reported that the retinal fluid only partially resolves in some patients after treatment [61,62]. Furthermore, as many as 10% of patients undergoing treatment exhibit a deterioration of symptoms [13,63,64]. It is the 20–40% of patients who may exhibit no response, and an additional subset who only partially respond, that necessitate alternative treatment strategies to optimize therapeutic outcomes. Due to the ambiguity surrounding these classifications, Amoaku et al. have endeavored to delineate non-responders and partial responders in nAMD based on both visual and anatomical responses [65]. Observing “non-responders” often raises the question of whether genetic variants could influence treatment response and consequently serve as biomarkers to discriminate or predict outcomes [66]. Previous studies have focused on the involvement of inflammation in both the development and progression of AMD. In particular, dysregulation of the complement system is an important factor in the pathogenesis of neovascular AMD. The *CFH* gene plays a significant role as an inhibitor of the complement cascade and has recently emerged as a key susceptibility gene for AMD. Studies investigating the genetics of AMD have identified susceptibility loci on chromosomes 1q31 and 10q26 [67], with the most compelling evidence of genetic risk associated with AMD observed in complement factor H (CFH) located on chromosome 1q31 [68]. CFH is composed of 20 modules of the complement control protein. The Y402H polymorphism (rs1061170) is located within a binding site for heparin and C-reactive protein, which has been implicated in the pathogenesis of AMD. Consequently, alterations in this specific region of the CFH protein may result in a dysfunctional CFH that is unable to adequately inhibit the complement cascade, potentially contributing to the pathophysiology of AMD. Wu et al. noted that the correlation strength between the rs1061170 polymorphism and AMD seems to diminish when studies transition from Western to Eastern populations. They found that the strong association observed in European cohorts did not translate to the same level of relevance for AMD risk in Asian ancestry populations [33]. KDR is a protein-coding gene that belongs to the VEGFR family and plays a critical role as a primary mediator of VEGF-induced processes such as endothelial proliferation, survival, migration, tubular morphogenesis, and sprouting. KDR is widely considered to play a crucial role in mediating VEGF-induced responses in angiogenesis [69]. Hagstrom et al. [70] studied patients with neovascular AMD who were genotyped for the SNP rs1061170 (CFH) to determine their response to treatment with ranibizumab or bevacizumab. In their study, no statistically significant differences in response by genotype were found for any of the clinical parameters analyzed. Specifically, no high-risk alleles were found to predict final visual acuity, changes in visual acuity, anatomical response (such as the presence of fluid on OCT or FA, retinal thickness, changes in total foveal thickness, or lesion size), or the number of injections required. Additionally, variations in response to anti-VEGF therapy were observed in terms of the frequency of injections needed. Five Genome-Wide Association Studies (GWAS) have been carried out, concerning the treatment of neovascular age-related macular degeneration (nAMD) [71,72,73,74,75], each comprising both a discovery and a replication cohort. However, none of these studies uncovered a variant with genome-wide significance. Notably, the *p*-values in the replication cohorts consistently showed less significance compared to the respective discovery studies, despite the replication cohort typically encompassing a larger sample size than the discovery study. The available data from meta-analyses allow a similar conclusion to be drawn, as the reported effects are often small or not reproducible. Of note, a study by Wang et al. [76] recalculated the results of 33 publications on genetic effects in response to anti-VEGF therapy in nAMD. In this study, SNP variants in nine genes were found to be significantly associated with response to anti-VEGF therapy; one of the samples NM is rs1410996 in the *CFH* gene. Further studies based on different ethnicities and large sample sizes are warranted to corroborate the findings found in the present study. The results of the ethnic subgroup analysis showed that rs800292-G and rs1061170-T in the CFH gene were associated with poor response to therapy in East Asians and Europeans, respectively. Although studies have attempted to characterize patients who either do not respond to anti-VEGF therapy or whose response declines over time [77], the mechanisms behind non-response and declining response remain unclear. The heterogeneity of response to anti-VEGF treatment in nAMD patients has led to increasing pharmacogenetic studies on the association of potential high-risk biomarkers associated with nAMD responding to anti-VEGF treatment.

## 5. Conclusions

CFH polymorphisms influence susceptibility to AMD but do not correlate with a response to anti-VEGF therapy. Further research is imperative to fully evaluate the developmental significance, treatment efficacy and predictive role in influencing susceptibility to anti-VEGF therapy for KDR and CFH.

## Figures and Tables

**Table 1:** Demographic data of the study.

*Characteristic*	*Early AMD* *n = 255*	*Exudative AMD* *n = 252*	*Control* *n = 349*	*p-Value*
Gender Males, n (%) Females, n (%)	81 (31.8) 174 (68.2)	94 (37.3) 158 (62.7)	121 (34.7) 228 (65.3)	0.455 * 0.507 **
Age years; median (IQR)	73 (12)	77 (10)	72 (11)	0.119 * <0.001 **

*p*—significance level, significance when *p* < 0.05; IQR—interquartile range; * early AMD vs. control group; ** exudative AMD vs. control group.

**Table 2:** PCR mixture.

Reagents	1 Sample	96 Samples
TaqMan Universal Master Mix II, no UNG (“Applied Biosystems”, Vilnius, Lithuania) “Applied Biosystems” genotyping assay C___8355565_10 C___2530294_10 C__15869271_10 C__11895315_20 H_2_O (“ZYMO RESEARCH”, Lithuania)	5 µL	480 µL
	0.5 µL	48 µL
	3.5 µL	336 µL

**Table 3:** RT-PGR reaction conditions for CFH and KDR genes’ polymorphism.

Gene, SNP	RT-PGR Reaction Conditions
CFH rs1061170 rs1410996	95 °C 10 min 45 cycles: 92 °C 15 s 60 °C 60 s
KDR rs2071559	
KDR rs1870377	

**Table 4:** Distributions of *KDR* and *CFH* SNPs genotypes and alleles in early, exudative AMD and control groups.

Gener/Marker	Genotype/ Allele	Group			*p*-Value *	*p*-Value **
		Early AMD (n = 255) n (%)	Exudative AMD (n = 252) n (%)	Control (n = 349) n (%)		
*KDR* rs2071559	GG GA AA G A	60 (23.2) 118 (46.3) 77 (30.2) 238 (46.7) 272 (53.3)	49 (19.4) 134 (53.2) 69 (27.4) 232 (46.0) 272 (54.0)	77 (22.1) 188 (53.9) 84 (24.1) 342 (49.0) 356 (51.0)	0.143 0.423	0.571 0.310
*KDR* rs1870377	TT TA AA T A	127 (49.7) 101 (39.6) 27 (10.6) 355 (69.6) 155 (30.4)	125 (49.6) 99 (39.3) 28 (11.1) 349 (69.2) 155 (30.8)	172 (49.3) 145 (41.5) 32 (9.2) 489 (70.1) 209 (29.9)	0.799 0.866	0.691 0.763
*CFH* rs1061170	TT TC CC T C	80 (31.4) 130 (51) 45 (17.6) 290 (56.9) 220 (43.1)	38 (15.1) 130 (51.6) 84 (33.3) 206 (40.9) 298 (59.1)	137 (39.3) 158 (45.3) 54 (15.5) 432 (61.9) 266 (38.1)	0.137 0.078	**<0.001** **<0.001**
*CFH* rs1410996	GG GA AA G A	114 (44.7) 108 (42.4) 33 (12.9) 336 (65.9) 174 (34.1)	162 (64.3) 82 (32.5) 8 (3.2) 406 (80.6) 98 (19.4)	132 (37.8) 168 (48.1) 49 (14) 432 (61.9) 266 (38.1)	0.232 0.155	**<0.001** **<0.001**

*p*—significance level, Bonferroni corrected significance level *p* = 0.05/8. * Early AMD vs. control group; ** exudative AMD vs. control group.

**Table 5:** Associations between *CFH* rs1061170 and early AMD, and *CFH* rs1061170 and rs1410996 with exudative AMD.

Genetic Model	Genotype/Allele	OR * (95 %CI)	*p*-Value	AIC
Early AMD				
*CFH* rs1061170				
Dominant	TC + CC vs. TT	1.414 (1.005; 1.988)	0.046	820.629
Exudative AMD				
*CFH* rs1061170				
Codominant	TC vs. TT CC vs. TT	2.961 (1.894; 4.630) 5.578 (3.319; 9.377)	**<0.001** **<0.001**	715.997
Dominant	TC + CC vs. TT	3.629 (2.372; 5.552)	**<0.001**	722.380
Recessive	CC vs. TT + TC	2.704 (1.796; 4.071)	**<0.001**	738.372
Additive	C	2.354 (1.820; 3.046)	**<0.001**	715.559
*CFH* rs1410996				
Codominant	GA vs. GG AA vs. GG	0.380 (0.262; 0.550) 0.119 (0.053; 0.266)	**<0.001** **<0.001**	712.575
Dominant	GA + AA vs. GG	0.318 (0.223; 0.453)	**<0.001**	719.925
Recessive	AA vs. GG + GA	0.184 (0.084; 0.403)	**<0.001**	737.824
Overdominant	GA vs. GG + AA	0.509 (0.357; 0.725)	**<0.001**	747.483
Additive	A	0.363 (0.270; 0.488)	**<0.001**	710.725

*—OR adjusted for age in exudative AMD group; OR—odds ratio; CI—confidence interval; *p*—significance level, Bonferroni corrected significance level *p* = 0.05/4; AIC—Akaike information criteria.

**Table 6:** Associations between *KDR* rs2071559, *CFH* rs1061170 and rs1410996 with early and exudative AMD in male and female groups.

Genetic Model	Genotype/Allele	OR * (95 %CI)	*p*-Value	AIC
Males				
Early AMD				
*KDR rs2071559*				
Codominant	GA AA	0.491 (0.254; 0.946) 0.657 (0.295; 1.463)	**0.033** 0.304	271.499
Dominant	GA + AA vs. TT	0.536 (0.290; 0.991)	**0.047**	270.093
Exudative AMD				
*KDR rs2071559*				
Codominant	GA AA	0.500 (0.266; 0.940) 0.531 (0.239; 1.181)	**0.031** 0.121	292.937
Dominant	GA + AA vs. TT	0.508 (0.279; 0.925)	**0.027**	290.964
*CFH* rs1061170				
Codominant	TC vs. TT CC vs. TT	3.698 (1.775; 7.704) 8.457 (3.442; 20.780)	**<0.001** **<0.001**	271.865
Dominant	TC + CC vs. TT	4.620 (2.278; 9.369)	**<0.001**	274.871
Recessive	CC vs. TT + TC	3.432 (1.687; 6.983)	**<0.001**	283.553
Additive	C	2.354 (1.820; 3.046)	**<0.001**	270.515
*CFH* rs1410996				
Codominant	GA vs. GG AA vs. GG	0.400 (0.223; 0.719) 0.036 (0.036; 0.278)	**0.002** **0.001**	270.037
Dominant	GA + AA vs. GG	0.302 (0.171; 0.532)	**<0.001**	277.719
Recessive	AA vs. GG + GA	0.053 (0.007; 0.406)	**0.005**	277.689
Overdominant	GA vs. GG + AA	0.566 (0.322; 0.995)	**0.048**	281.94\
Additive	A	0.314 (0.193; 0.510)	**<0.001**	270.282
Females				
Exudative AMD				
*CFH* rs1061170				
Codominant	TC vs. TT CC vs. TT	2.608 (1.470; 4.629) 4.476 (2.346; 8.539)	**0.001** **<0.001**	438.402
Dominant	TC + CC vs. TT	3.163 (1.843; 5.427)	**<0.001**	440.214
Recessive	CC vs. TT + TC	2.399 (1.440; 3.996)	**<0.001**	447.718
Additive	C	2.102 (1.562; 2.895)	**<0.001**	437.210
*CFH* rs1410996				
Codominant	GA vs. GG AA vs. GG	0.359 (0.221; 0.583) 0.182 (0.072; 0.460)	**<0.001** **<0.001**	434.805
Dominant	GA + AA vs. GG	0.321 (0.202; 0.511)	**<0.001**	434.974
Recessive	AA vs. GG + GA	0.291 (0.119; 0.713)	**0.007**	450.722
Overdominant	GA vs. GG + AA	0.464 (0.292; 0.736)	**<0.001**	448.285
Additive	A	0.392 (0.269; 0.573)	**<0.001**	433.132

*—OR adjusted for age in exudative AMD group; OR—odds ratio; CI—confidence interval; *p*—significance level, Bonferroni corrected significance level *p* = 0.05/4; AIC—Akaike information criteria.

**Table 7:** Linkage disequilibrium between *KDR* and *CFH* SNPs.

SNPs	*D’*	*r* ^2^
	Early AMD vs. Controls	
rs2071550-rs1870377	0.2282	0.0207
rs1061170-rs1410996	0.8068	0.2510
	**Exudative AMD vs. Controls**	
rs2071550-rs1870377	0.2341	0.0216
rs1061170-rs1410996	0.7954	0.2430

SNPs—single nucleotide polymorphisms; *D’* is the deviation between the expected haplotype frequency and the observed frequency [*D*’ scale: 0,1]. *r*^2^ is the squared correlation coefficient of the haplotype frequencies [*r*^2^ scale: 0,1]; *p*—significance level, significant when *p* < 0.05.

**Table 8:** *KDR* and *CFH* haplotype association with AMD.

Haplotype	rs2071559	rs1870377	rs1061170	rs1410996	Frequency	OR * (95% CI)	*p*-Value
Haplotype associations with early AMD							
1	G	T	-	-	0.3692	1.00	-
2	A	T	-	-	0.3295	1.24 (0.91–1.69)	0.17
3	A	A	-	-	0.1904	1.05 (0.76–1.46)	0.77
4	G	A	-	-	0.1109	1.28 (0.80–2.03)	0.3
5	-	-	C	G	0.3737	1.00	-
6	-	-	T	A	0.3357	0.76 (0.58–1.00)	**0.049**
7	-	-	T	G	0.2620	0.60 (0.45–0.82)	**0.0011**
Haplotype associations with exudative AMD							
1	G	T	-	-	0.3673	1.00	-
2	A	T	-	-	0.3298	1.28 (0.93–1.77)	0.13
3	A	A	-	-	0.1926	1.10 (0.78–1.53)	0.59
4	G	A	-	-	0.1102	1.28 (0.81–2.03)	0.3
5	-	-	C	G	0.4402	1.00	-
6	-	-	T	A	0.2738	0.29 (0.20–0.40)	**<0.001**
7	-	-	T	G	0.257	0.42 (0.30–0.58)	**<0.001**
8	-	-	C	A	0.0287	0.03 (0.00–0.21)	**<0.001**

*—OR adjusted for age in exudative AMD group; s; OR—odds ratio; CI—confidence interval; *p*—significance level *p* < 0.05.

**Table 9:** Demographic and clinical parameter.

Characteristic	Non-Responders n = 22	Responders n = 99	*p*-Value
Gender	7 (31.8) 15 (68.2)	32 (32.3) 67 (67.7)	0.963
Males, n (%)			
Females, n (%)			
Age years; median (IQR)	77 (10)	78 (10)	0.184
Response parameter			
VA, median (IQR)			
Baseline	0.42 (0.45)	0.26 (0.26)	0.020 *
Treated(final)	0.31 (0.31)	0.35 (0.32)	<0.001 **
CRT (μm), median (IQR)			
Baseline	272.5 (86.5)	321 (114)	0.386 *
Treated (final)	314.5 (87.75)	274 (94)	<0.001 **

*p*—significance level, significance when *p* < 0.05; IQR—interquartile range; VA—visual acuity; CRT—central macular thickness; * non-responders: baseline vs. treated; ** responders: baseline vs. treated.

## Data Availability

The datasets used and/or analyzed during the current study are available from the corresponding author on reasonable request.
